# Genome-Wide Association Study Reveals a Polymorphism in the Podocyte Receptor RANK for the Decline of Renal Function in Coronary Patients

**DOI:** 10.1371/journal.pone.0114240

**Published:** 2014-12-05

**Authors:** Andreas Leiherer, Axel Muendlein, Philipp Rein, Christoph H. Saely, Elena Kinz, Alexander Vonbank, Peter Fraunberger, Heinz Drexel

**Affiliations:** 1 Vorarlberg Institute for Vascular Investigation and Treatment (VIVIT), Feldkirch, Austria; 2 Department of Medicine and Cardiology, Academic Teaching Hospital Feldkirch, Feldkirch, Austria; 3 Private University of the Principality of Liechtenstein, Triesen, Liechtenstein; 4 Medical Central Laboratories, Feldkirch, Austria; 5 Drexel University College of Medicine, Philadelphia, PA, United States of America; University of Utah School of Medicine, United States of America

## Abstract

Impaired kidney function is a significant health problem and a major concern in clinical routine and is routinely determined by decreased glomerular filtration rate (GFR). In contrast to single assessment of a patients' kidney function providing only limited information on patients' health, serial measurements of GFR clearly improves the validity of diagnosis. The decline of kidney function has recently been reported to be predictive for mortality and vascular events in coronary patients. However, it has not been investigated for genetic association in GWA studies. This study investigates for the first time the association of cardiometabolic polymorphisms with the decline of estimated GFR during a 4 year follow up in 583 coronary patients, using the Cardio-Metabo Chip. We revealed a suggestive association with 3 polymorphisms, surpassing genome-wide significance (p = 4.0 e-7). The top hit rs17069906 (p = 5.6 e-10) is located within the genomic region of RANK, recently demonstrated to be an important player in the adaptive recovery response in podocytes and suggested as a promising therapeutic target in glomerular diseases.

## Introduction

Glomerular disease is defined by dysfunction, injury, or loss of podocytes [1]. Genome wide association (GWA) studies have identified several loci associated with estimated glomerular filtration rate [2], [3], accounting only for 1.4% of estimated glomerular filtration rate (eGFR) variation [2]. Critical values are usually tracked over a longer period in high risk patients affected with coronary diseases or diabetes. We recently proposed that a dynamic decrease in eGFR may act as a noninvasive marker for the increase of microangiopathy and the progression of atherosclerotic disease in the renal vasculature [4]. In this context, serial measurements of eGFR appears preferable to only one single assessment of kidney function, providing only limited information on patients' health. Moreover, the decline of kidney function is also predictive for mortality and vascular events [4], [5].

The serial decline of kidney function has not been investigated for genetic association in GWA studies so far. This study is the first to aim at investigating genotypes associated with renal decline in a high-risk population of coronary patients.

## Results

### Patient characteristics

A population of high-risk patients for atherosclerotic disease was built in which coronary atherosclerosis was directly proven and quantified by visualization in coronary arteriography. The baseline characteristics of our study population (n = 583) were thus typical for a cohort undergoing angiography for the evaluation of coronary artery disease (CAD), with a mean age of 64 years, a preponderance of male gender (65%) and a high prevalence of type 2 diabetes mellitus (T2DM; 22%). A total of 55% had significant CAD at angiography, and the mean baseline eGFR was 98.6 ml/min/1.73 m^2^. After a mean (±SD) follow-up time of 3.3±0.5 years, eGFR was reassessed in these 583 subjects. We noticed a mean decrease of 4.9 ml/min/1.73 m2. Thereupon, the study cohort was separated in two groups according to the median of decrease of eGFR during the follow up time. For comparison, the cohort was also separated by the median eGFR from a single measurement at baseline.

During the follow up time, we recorded 105 non-fatal vascular events in 80 patients, encompassing 15 non-fatal myocardial infarctions, 13 non-fatal ischemic strokes, 15 coronary artery bypass graft (CABG) surgeries, 37 percutaneous coronary interventions (PCIs), and 25 non-coronary revascularizations at the carotid and peripheral arteries. First vascular events occurred in 13.7% of the study population, amounting to an annual event rate of 4.1%.

### Glomerular filtration rate and cardiovascular events

Table 1 summarizes the study characteristics of our patients with respect to differences between baseline eGRF measurement and the decline of eGFR over time. Of note, we assessed that a high decrease of eGFR was significantly associated with cardiovascular events (p = 0.032), whereas a high baseline eGFR was not (p = 0.480). Similarly, the decline of eGFR, used as a continuous variable, was a significant predictor of vascular events with a hazard ratio of 1.24 ([95%CI 1.03–1.48], p = 0.021), whereas eGFR from single measurement did not predict these events (HR = 1.09 [95%CI 0.87–1.37], p = 0.450). We also recognized that patients with CAD at baseline had a significantly more pronounced decline than those without CAD (5.8 vs. 3.8 ml/min/1.73 m2, p = 0.001), but there was no significant difference with respect to baseline eGFR (99.3 vs. 97.8 ml/min/1.73 m^2^, p = 0.151).

**Table 1:** Patient characteristics according to baseline eGFR and decline of eGFR.

	total (n = 583)			low eGFR (n = 291)			high eGFR (n = 292)			p-value	low ΔeGFR (n = 291)			high ΔeGFR (n = 292)			p-value
Age (years)	64	±	10	68	±	8	60	±	11	<0.001	66	±	9	63	±	11	0.031
Male sex (%)	65%			37%			93%			<0.001	52%			79%			<0.001
BMI (kg/m2)	27.6	±	4.1	27.8	±	4.5	27.5	±	3.8	0.448	27.4	±	4.0	27.9	±	4.3	0.426
CAD (%)	55%			49%			60%			0.014	47%			62%			<0.001
Extent of CAD	1.3	±	1.7	1.2	±	1.7	1.3	±	1.6	0.136	1.1	±	1.7	1.4	±	1.7	0.003
Vascular events (%)	14%			13%			15%			0.480	11%			17%			0.032
T2DM (%)	22%			22%			22%			0.982	20%			24%			0.205
Smoking (%)	58%			45%			71%			<0.001	52%			64%			0.004
eGFR (ml/min/1.73 m2)	98.6	±	17.1	85.3	±	12.5	111.9	±	8.7	<0.001	93.5	±	14.8	##	±	17.7	<0.001
ΔeGFR (ml/min/1.73 m2)	4.9	±	11.6	3.6	±	12.4	6.1	±	10.7	<0.001	−1.5	±	6.7	11.3	±	12.0	<0.001
Triglycerides (mg/dl)	140	±	87	131	±	73	149	±	98	0.299	132	±	70	148	±	100	0.675
Total cholesterol (mg/dl)	197	±	46	197	±	45	196	±	46	0.925	196	±	48	197	±	44	0.620
LDL cholesterol (mg/dl)	128	±	41	127	±	40	130	±	43	0.344	128	±	42	129	±	41	0.694
HDL cholesterol (mg/dl)	57	±	17	60	±	19	54	±	15	<0.001	58	±	18	56	±	16	0.527
Fasting glucose(mg/dl)	104	±	32	102	±	26	106	±	37	0.755	102	±	26	107	±	37	0.427
Systolioc blood pressure (mm Hg)	137	±	17	138	±	18	135	±	17	0.018	136	±	17	137	±	17	0.505
Diastolioc blood pressure (mm Hg)	82	±	9	82	±	9	82	±	10	0.382	82	±	9	82	±	9	0.198
CRP (mg/dl)	0.38	±	0.67	0.38	±	0.61	0.37	±	0.73	0.351	0.36	±	0.57	0.39	±	0.76	0.957
Fibrinogen (mg/dl)	327	±	67	336	±	67	317	±	65	0.001	330	±	69	323	±	64	0.426
ACE inhib./AT-2 antag. treatment (%)	40%			42%			38%			0.294	38%			41%			0.520
Statin treatment (%)	50%			50%			49%			0.836	48%			51%			0.431
Biguanide treatment (%)	9%			9%			10%			0.577	7%			11%			0.089

Patients have been assigned to two groups by the median of eGFR at baseline (99.19 ml/min/1.73 m^2^.) and in contrast by the median of eGFR decline (or change, respectively) between baseline and the 3.5 year follow up (ΔeGFR; 2.39 ml/min/1.73 m^2^). All data are given as means ± standard deviation or percentages and p-values each express the difference between respective two groups. CAD is defined by angiographically determined coronary artery stenoses with lumen narrowing ≥50%. CAD denotes coronary artery disease, BMI body mass index, T2DM type 2 diabetes mellitus, CRP C-reactive protein, LDL low density lipoprotein, HDL high density lipoprotein, ACE angiotensin converting enzyme, and AT-2 angiotensin 2.

### Genome wide analysis

**Figure 1:**
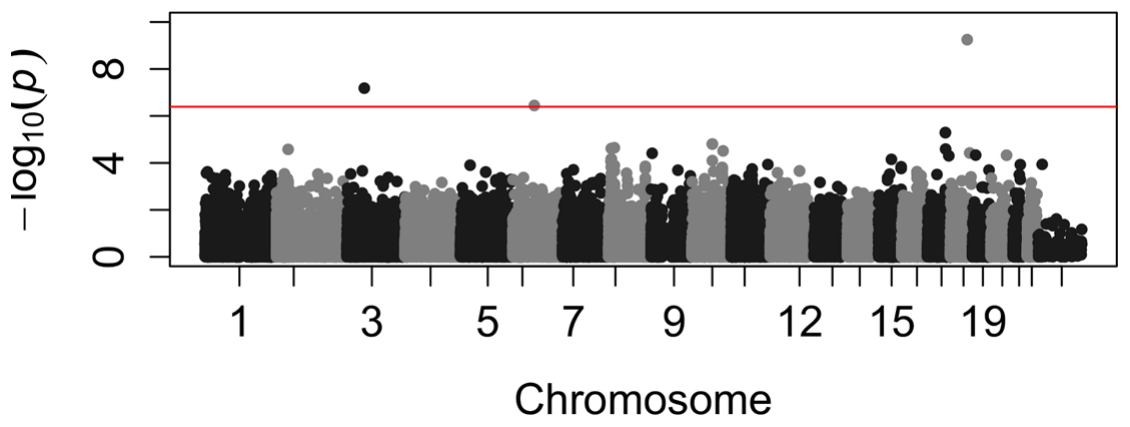
Manhattan plot of genome-wide association study of decline of eGFR. SNPs were characterized using the Illumina Cardio-Metabo Chip. After frequency and genotyping pruning, there were 124215 SNPs left. Highest association was seen in rs17069906 (p = 5.63 e-10), rs9812824 (p = 6.53 e-8), and rs9588431 (p = 3.57 e-7).The red line indicates genome-wide significant associations (4.02 e-7).SNPs are associated with decline of eGFR independently from baseline eGFR.

Analyzing our 583 individual patient samples we did not observe genomic inflation (λ = 1.02, figure S1). Results of the GWA regarding the decline of eGFR are presented in figure 1. We revealed significant genome-wide associations between single nucleotide polymorphisms (SNPs) rs17069906 (nominal p-value = 5.6 e-10), rs9812824 (nominal p-value = 6.5 e-8), and rs9688431 (nominal p-value = 3.6 e-7) and the decline of eGFR even if applying stringent criteria (Bonferroni corrected p-value = 7.0 e-5, 8.1 e-3, and 4.4 e-2 respectively). Polymorphism rs17069906 is located in the intronic region of the NFκB activator (RANK), also known as tumor necrosis factor receptor superfamily, member 11a, NFκB activator (TNFRSF11A), at chromosome 18 and was the top hit in the GWA. The second SNP rs9812824 lies in a non-coding region at locus 3p14.2, 158 kb downstream the gene for fragile histidine triad (FHIT) and separated by 55 kb from small nuclear ribonucleoprotein polypeptide B pseudogene (LOC 100421672). The third SNP rs9688431 is located on chromosomal locus 6q13, 14 kb downstream the gene for potassium voltage-gated channel KQT-like subfamily member 5 (KCNQ5).

### SNPs are associated with decline of eGFR independently from baseline eGFR

Underlining the discrimination between decline of eGFR and baseline eGFR, we assessed no significant association of baseline eGFR with rs17069906 (β = −0.62, p = 0.137), rs9688431, (β = 0.27, p = 0.522), and rs9812824 (β = 0.79, p = 0.055). In covariance analysis, after adjustment for age, gender, body mass index, presence of type 2 diabetes as well as baseline eGFR, the top hit rs17069906 (F = 38.9, p = 8.6 e-10) as well as polymorphisms rs9812824 (F = 30.8, p = 4.4 e-8) and rs9688431 (F = 24.3, p = 1.1e-6) were significantly associated with the decline of eGFR and, in case of rs17069906 and rs9812824, even passed Bonferroni correction. Moreover, the interaction term baseline eGFR by decline of eGFR was not significant with regard to rs1706906 (p = 0.485), implicating no impact of baseline eGFR on the association between decline of eGFR and rs17069906. Similarly, the same interaction term was not significant if testing association between decline of eGFR and rs9688431 (p = 0.396), but proved to be significant in case of rs9812824 (p = 1.1e-4).

Comparing the highest decline of eGFR to the lowest decline after categorization (p = 0.003) as well as the trend in groups (p_trend_ = 0.009) confirmed the relationship between rs17069906 and the change in eGFR after 3.3 years for five groups and similar results are seen for four groups (p = 0.016, p_trend_ = 0.013).

### Polymorphisms as predictors of cardiovascular events

Variation rs9812824 proved significantly predictive of cardiovascular events with a hazard ratio of 3.32 (95%CI 1.21–9.08, p = 0.019). After adjustment for age, gender, BMI, and type 2 diabetes, rs9812824 still significantly predicted cardiovascular events (adjusted HR = 3.25 [95%CI 1.17–9.02], p = 0.024]. Of note, it still impacted the risk for future events after additional adjustment for baseline eGFR (adjusted = HR 3.19 [95%CI 1.15–8.89], p = 0.026) but not if adjusting for decline of eGFR instead of baseline eGFR (adjusted HR = 2.13 [95%CI 0.66–6.88], p = 0.204). In contrast, there was no association between rs17069906 or rs9688431 and the risk for cardiovascular events (unadjusted HR = 0.83 [95%CI 0.26–2.62], p = 0.747 and unadjusted HR = 0.78 [95%CI 0.38–1.61], p = 0.505).

### Interrogation of known renal function loci for their association with eGFR and decline of eGFR

In previous studies addressing the genome-wide association with renal function, SNPs have been identified in genes including UMOD, SHROOM3, STC1, ALMS1, DAB2, SLC34A1, PRKAG2, VEGFA, DACH1, and SLC7A9 [2], [3], [6], [7]. Given the metabolic and atherosclerotic-cardiovascular focus, the Cardio-Metabo Chip does not cover all previously identified SNPs. Nevertheless, 8 SNPs, previously demonstrated to be in association with eGFR [2], [3], [6], [7], were present on the Cardio-Metabo Chip and 4 additional ones were perfectly or highly linked with those previously described (table 2). Testing the association between these SNPs and eGFR values, using a previously described cohort of 1629 patients [8], SNPs rs7422339 representing CPS1, rs10224210 representing PRKAG2, and rs4744712 representing PIP5K1B proved to be significant (p = 0.029, p = 0.008, and p = 0.005 respectively) in linear regression analysis, and rs17319721 representing SHROOM3, rs9472138 representing VEGFA, and rs8101881 representing SLC7A9 only slightly missed the level of significance (p = 0.052, p = 0.061, and p = 0.079 respectively; table 2). In contrast, if using the decline of eGFR instead of eGFR, none of these SNPs reached statistical significance in our patients.

**Table 2:** Interrogation of known loci for association with eGFR.

SNP	Chr.	Position	Alternate SNP	Position	r2	Genes	β	p-value
rs17319721	4	77587871	-	-	-	SHROOM3	−0.049	0.052
rs267734	1	149218101	-	-	-	ANXA9	0.013	0.616
rs1260326	2	27584444	-	-	-	GCKR	0.009	0.720
rs653178	12	110492139	-	-	-	ATXN2	−0.006	0.797
rs12917707*	16	*20275191*	rs12922822	20275146	1	UMOD	−0.013	0.594
rs881858*	6	*43914587*	rs9472138	43919740	0.73	VEGFA	0.047	0.061
rs2279463	6	160588379	-	-	-	SLC22A2	−0.021	0.406
rs10224210	7	151044127	-	-	-	PRKAG2	−0.067	0.008
rs4744712	9	70624527	-	-	-	PIP5K1B	−0.071	0.005
rs12460876*	19	*38048731*	rs8101881	38056468	1	SLC7A	0.044	0.079
rs7422339	2	211248752	-	-	-	CPS1	−0.055	0.029
rs6465825*	7	*77254375*	rs1544459	77255520	0.79	TMEM60	0.021	0.400

*Positions are given according to NCBI 36.3 genome build. SNPs were assigned to genes within 60 kb. Alternate SNPs were used for SNPs lacking on the Cardio-Metabo Chip (asterisks) if in high LD (r^2^<0.7). Association is given as β coefficient and p-value according to linear regression analysis.*

## Discussion

In this GWA study, we for the first time show significant associations of a polymorphism in RANK with the decline of eGFR in a high risk cohort of coronary patients. Moreover, also a significant association for two polymorphisms in non-coding regions located near FHIT and close to KCNQ5 exists.

Podocytes are highly specialized cells with an important role in maintaining the glomerular filtration barrier [9], lining the outer surface of the glomerular basement membrane [10]. Podocyte injury or apoptosis result in a damaged glomerular filtration barrier and renal dysfunction. However, there are several repair mechanisms determining the fate of podocytes in response to injury [9]. One of these is RANK and its ligand RANKL, known as important regulators of cell interaction [11] and to be involved in cell survival and apoptosis [12]. Of note, RANK has been recently proposed as a part of an adaptive recovery response to podocyte injury by Liu et al [12]. In animal models RANK gene expression as well as protein levels in the glomerular walls were significantly increased after podocyte injury [13]. Concordantly, in patients with proven kidney disease encompassing focal segmental glomerulosclerosis, IgA nephropathy, membranous nephropathy, RANK expression is significantly increased [13]. Thus, due to its ability to inhibit apoptosis, RANK has been suggested to play a pivotal role in the pathogenesis of podocyte injury [13]. Our association data, linking the decline of glomerular filtration to a RANK polymorphism, are corroborating the new role of RANK and RANKL in podocyte repair and clearly support its link to renal dysfunction.

Proceeding from our data supporting the link between RANK and glomerular disease, further attempts seem necessary to elucidate the biological background of SNPs near KCNQ5 and FHIT for renal impairment. The former, a potassium channel gene setting the membrane potential in epithelia [14], is a candidate gene for autism [15] and has been associated with disc calcification in dachshund [16] and refractive error of the eye in human [17]. However, we did not find a convincing biological link between renal decline and associated regions nearby KCNQ5. One may speculate whether this region contains regulatory elements controlling the expression of a causal gene located outside the identified region. Further functional and epidemiological studies targeting that locus may clarify its association with renal decline.

Variant rs9812824 is located in the common fragile site 3p14.2, the most active common fragile site in the human genome [18] downstream the FHIT gene. FHIT is a tumor suppressor, appearing necessary for DNA damage response [19]. Genetic alteration of FHIT and nearby genomic region and its involvement in renal disease (carcinoma) is very well documented [20]–[26]. Of note, formal interaction analysis also revealed a significant interaction between baseline eGFR and the decline of eGFR in covariance analysis. This indicates a significant impact of baseline filtration rate on the association between the decline of renal function and rs9812824, which, however, does not apply for rs7069906 and rs9688431, suggesting a different functional background. Thus the polymorphism's association with renal decline together with the predictive power for cardiovascular events might indicate a modulator role of that genomic region for kidney disease.

Previously, we have proposed that a rapid decline of eGFR is a predictor for death and vascular events [4], [27]. Of note, it has also been demonstrated that the decline of eGFR is an independent marker for these events and that eGFR at baseline did not significantly impact the association of decline of eGFR with the mortality risk [4]. In accordance, we demonstrated in a formal interaction analysis that the interaction term baseline eGFR by decline of eGFR is not significant (p = 0.205) and, therefore, does not modulate the association between the RANK polymorphism rs17069906 and the decline of eGFR in analysis of covariance. This supports the usefulness of serial decline of eGFR as an independent prognostic marker.

In addition, the percentage of vascular events as well as the extent of CAD was significantly higher in patients with a high decline of eGFR compared to those with a low decline. Comparing patients with high and low baseline eGFR, we did not see such a significant difference. On the other hand, HDL cholesterol, systolic blood pressure, and fibrinogen only significantly differed with respect to high vs. low eGFR baseline but not if comparing high and low decline of eGFR.

Furthermore, from our replication data regarding previously described loci associated with eGFR, we were able to reproduce at least in part the association between these loci and baseline eGFR. However, these markers were not associated with the decline in renal function over the follow up time.

This illustrates the independent value of decline of eGFR in our GWA study extending the scope of recent data obtained solely by simply detecting the presence of renal dysfunction. Moreover, it underlines the value of assessing the decline of eGFR for prognosis in high risk coronary patients.

Several loci have been identified by genome-wide association studies to be associated with eGFR but this study is the first assessing genetic variations implicated in the decline of eGFR. The identification of variants biologically linked to glomerular disease and renal decline as described above is a particular strength of the study. Another important strength was the minimal population stratification (genomic inflation factor lambda = 1.02). Furthermore, the study also benefits from its very well characterized population as well as the long follow up time of 3.3 years.

This study has some limitations, so it deserves emphasizing that there are possible confounding effects with respect to RANK-RANKL signaling, especially in patients with cardiovascular disease. Vascular calcification is associated with atherosclerosis, diabetes, as well as kidney disease [28], and RANK, RANKL, as well as osteoprotegerin [29], may be important players in that process, although their detailed role is currently under investigation [30], [31].

Our study participants are a selected group and thus do not reflect the general population. They were patients undergoing coronary angiography for the evaluation of CAD and are also characterized by a higher prevalence of T2DM with 22% compared to 6.6% or 6.9% in the general Caucasian population of the surrounding area in Switzerland [32] or Germany [33]. However, eGFR, as far as it cold be compared, does not markedly differ between our cohort and data for the general population in literature [33]–[35]. Nevertheless, these study subjects deserve particular clinical interest as they represent a patient cohort with a high risk for cardiovascular events. The sample size of our study cohort appears moderate compared to previous studies or pooled analyses of existing data for the glomerular filtration rate and renal disease. However, data for serial renal decline are sparse and thus our modestly-sized study provides important findings as it is the first prospective study addressing the decline of glomerular filtration for genome wide association. Moreover, it should be stressed that the impact of the proposed associations is of high statistical significance and survives correlation for multiple testing according to Bonferroni. Nevertheless confirmation of these results with additional clinical data for the decline of eGFR in larger populations is necessary and may provide further putative associations, and in vitro studies should be performed to elucidate the functionality of these variants.

In conclusion, this study for the first time assessed the genome-wide association of genetic polymorphisms and the decline of eGFR over a 3.3 year follow up and thereby revealed a variation in the glomerular disease target, podocyte receptor RANK.

## Methods

### Study settings

From September 2005 through April 2008 Caucasian patients who were referred to elective coronary angiography for the evaluation of established or suspected stable CAD were consecutively recruited for a 4 year follow up study. Patients undergoing coronary angiography for other reasons were not enrolled. In particular, no patients with acute coronary syndromes were enrolled. In 644 patients, the renal status has been successfully assessed at baseline and follow up visit and from these, 583 individual samples passed the data quality control for statistical analysis. The present study has been approved by the Ethics Committee of the University of Innsbruck. Written informed consent was given by all participants.

### Study patients and laboratory analysis

Coronary angiography was performed with the Judkin's technique and the severity of stenosis was assessed by visual inspection by a team of two investigators who were blinded to serologic assays as described previously [36]. In short, coronary artery stenoses with lumen narrowing ≥50% were considered significant and the extent of CAD was defined as the number of significant coronary stenoses in a given patient. Coronary arteries were defined as normal in the absence of any visible lumen narrowing at angiography. Information on conventional cardiovascular risk factors was obtained by a standardized interview. Systolic/diastolic blood pressure was measured by the Riva–Rocci method under resting conditions in a sitting position at the day of hospital entry at least 5 h after hospitalization. Hypertension was defined according to the Seventh Report of the Joint National Committee on Prevention, Detection, Evaluation, and Treatment of High Blood Pressure [37], and type 2 diabetes mellitus (T2DM) was diagnosed according to World Health Organization criteria [38]. Height and weight were recorded, and body mass index (BMI) was calculated as body weight (kg)/height (m^2^). All non-fatal cardiovascular events have been recorded during the follow up time comprising myocardial infarction, ischemic stroke, and the need for aorto coronary bypass, percutaneous transluminal coronary angioplasty, or vascular surgery revascularization in the carotid or peripheral arterial beds. Revascularization procedures which have been planned as a consequence of the baseline examination were not regarded as future events.

Venous blood samples were collected after an overnight fast of 12 h before angiography was performed and laboratory measurements were performed from fresh plasma samples, as described previously [39]. Serum triglycerides, total cholesterol, low density lipoprotein (LDL) cholesterol, and high density lipoprotein (HDL) cholesterol were determined on a Hitachi 717 or 911 or a Cobas Integra 8000 (Roche, Basel, Switzerland).

### Renal status assessment

In this study, we successfully assessed the renal status at baseline and after a mean follow-up period of 3.3±0.5 years in 644 patients. The glomerular filtration rate (GFR) has been estimated according to the quadratic Mayo Clinic equation, which gives more accurate estimates of GFR in patients with nearly normal renal function [40]. If serum creatinine was <0.8 mg/dL, 0.8 mg/dL was inserted as a value for serum creatinine, as described previously [41].

### Quality control and preprocessing of Cardio- Metabo Chip data

Single Nucleotide Polymorphisms (SNPs) in cardiovascular and metabolism genes were characterized using the Illumina Cardio-Metabo Chip technology (Illumina Inc, San Diego, CA, USA). This chip is a high-density custom array that captures DNA variations at regions identified to be relevant for metabolic and atherosclerotic-cardiovascular traits and respective diseases, as described in Voight et al. [42]. Respective DNA from patient blood samples has been extracted using the chemagen magnetic separation module I (PerkinElmar Inc., Baesweiler, Germany) by magnetic beads based method from 2ml whole blood following the manufacturers manual and quantification of extracted nucleotides has been checked spectrophotometrically (NanoDrop; Thermo Scientific, Wilmington, DE, USA). Cardio-Metabo Chip read-out was performed at the Institute of Human Genetics, University of Bonn at the Life&Brain Center (Bonn, Germany). Analyzing the obtained data, we excluded patient samples if they had genotyping failure for more than 5%. In addition, samples identified to be derived from twins or from first grade related patients were removed. After quality control and exclusion of failing samples, there were 583 individual patient samples left for analysis. From a total of 196,725 SNPs those were excluded with a minor allele frequency less than 1% of the study population. In addition, SNPs were also excluded, if they failed to be genotyped in more than 1% of the study population or, if the SNP was not in Hardy-Weinberg equilibrium among samples (critical *p*-value = 1e-6). After frequency and genotyping pruning, there were 124,215 SNPs left. With these settings, we did not observe significant genomic inflation (λ = 1.02).

### Statistical analysis

Differences in baseline characteristics were tested for statistical significance with the Chi-squared tests for categorical and Mann-Whitney U tests for continuous variables, respectively. Correlation analyses were performed calculating non-parametric Spearman rank correlation coefficients. In addition, analysis of covariance models (ANCOVA) were built using a general linear model approach. Adjusted hazard ratios for the incidence of vascular events were derived from Cox proportional hazards models after z-transformation of continuous variables. Results are given as mean (standard deviation) if not denoted otherwise and p-values <0.05 were considered significant.

Bonferroni correction was used to adjust for the critical p-value threshold (p-value = 4.02e-7) accounting for multiple testing in overall genome-wide level analysis. Conducting secondary data analysis for evaluating associations between renal function and loci already described in literature and those also represented by the Cardio-Metabo Chip a threshold of 0.05 was used to determine statistical significance. All statistical analyses were performed with SPSS 20.0 for Windows (SPSS Inc., Chicago, IL, USA) and PLINK version 1.07 [43]; http://pngu.mgh.harvard.edu/purcell/plink/).

## Supporting Information

Figure S1 **Q-Q plot showing the absence of inflation.**
(TIF)

## References

[1] Wiggins RC (2007) The spectrum of podocytopathies: a unifying view of glomerular diseases. Kidney Int. 71:1205–1214. 17410103 10.1038/sj.ki.5002222

[2] Koettgen A, Pattaro C, Boger CA, Fuchsberger C, Olden M, et al (2010) New loci associated with kidney function and chronic kidney disease. Nat.Genet 42:376–384. 20383146 10.1038/ng.568 PMC2997674

[3] Koettgen A, Glazer NL, Dehghan A, Hwang SJ, Katz R, et al (2009) Multiple loci associated with indices of renal function and chronic kidney disease. Nat.Genet 41:712–717. 19430482 10.1038/ng.377 PMC3039280

[4] Rein P, Saely CH, Vonbank A, Boehnel C, Drexel H (2014) Usefulness of serial decline of kidney function to predict mortality and cardiovascular events in patients undergoing coronary angiography. Am.J.Cardiol 113:215–221. 24169005 10.1016/j.amjcard.2013.08.032

[5] Al Aly Z, Zeringue A, Fu J, Rauchman MI, McDonald JR, et al (2010) Rate of kidney function decline associates with mortality. J.Am.Soc.Nephrol 21:1961–1969. 20947634 10.1681/ASN.2009121210 PMC3014010

[6] Liu CT, Garnaas MK, Tin A, Kottgen A, Franceschini N, et al (2011) Genetic association for renal traits among participants of African ancestry reveals new loci for renal function0. PLoS.Genet 7:e1002264. 21931561 10.1371/journal.pgen.1002264 PMC3169523

[7] Chambers JC, Zhang W, Lord GM, van der HP, Lawlor DA, et al (2010) Genetic loci influencing kidney function and chronic kidney disease. Nat.Genet 42:373–375. 20383145 10.1038/ng.566 PMC3748585

[8] Muendlein A, Stark N, Rein P, Saely CH, Geller-Rhomberg S, et al (2012) Are AHSG polymorphisms directly associated with coronary atherosclerosis? Clin.Chim.Acta 413:287–290. 22024217 10.1016/j.cca.2011.10.008

[9] Leeuwis JW, Nguyen TQ, Dendooven A, Kok RJ, Goldschmeding R (2010) Targeting podocyte-associated diseases. Adv.Drug Deliv.Rev. 62:1325–1336. 20828590 10.1016/j.addr.2010.08.012

[10] Abrahamson DR (1987) Structure and development of the glomerular capillary wall and basement membrane. Am.J.Physiol 253:F783–F794. 3318497 10.1152/ajprenal.1987.253.5.F783

[11] Anderson DM, Maraskovsky E, Billingsley WL, Dougall WC, Tometsko ME, et al (1997) A homologue of the TNF receptor and its ligand enhance T-cell growth and dendritic-cell function. Nature 390:175–179. 9367155 10.1038/36593

[12] Bharti AC, Aggarwal BB (2004) Ranking the role of RANK ligand in apoptosis. Apoptosis. 9:677–690. 15505411 10.1023/B:APPT.0000045780.10463.c6

[13] Liu S, Shi W, Xiao H, Liang X, Deng C, et al (2012) Receptor activator of NF-kappaB and podocytes: towards a function of a novel receptor-ligand pair in the survival response of podocyte injury. PLoS.One 7:e41331. 22848465 10.1371/journal.pone.0041331 PMC3405116

[14] Robbins J (2001) KCNQ potassium channels: physiology, pathophysiology, and pharmacology. Pharmacol.Ther. 90:1–19. 11448722 10.1016/s0163-7258(01)00116-4

[15] Gilling M, Rasmussen HB, Calloe K, Sequeira AF, Baretto M, et al (2013) Dysfunction of the Heteromeric KV7.3/KV7.5 Potassium Channel is Associated with Autism Spectrum Disorders. Front Genet. 4:54. 23596459 10.3389/fgene.2013.00054 PMC3627139

[16] Mogensen MS, Scheibye-Alsing K, Karlskov-Mortensen P, Proschowsky HF, Jensen VF, et al (2012) Validation of genome-wide intervertebral disk calcification associations in dachshund and further investigation of the chromosome 12 susceptibility locus. Front Genet. 3:225. 23125846 10.3389/fgene.2012.00225 PMC3485664

[17] Verhoeven VJ, Hysi PG, Wojciechowski R, Fan Q, Guggenheim JA, et al (2013) Genome-wide meta-analyses of multiancestry cohorts identify multiple new susceptibility loci for refractive error and myopia. Nat.Genet. 45:314–318. 23396134 10.1038/ng.2554 PMC3740568

[18] Smeets DF, Scheres JM, Hustinx TW (1986) The most common fragile site in man is 3p14. Hum.Genet. 72:215–220. 2937710 10.1007/BF00291880

[19] Okumura H, Ishii H, Pichiorri F, Croce CM, Mori M, et al (2009) Fragile gene product, Fhit, in oxidative and replicative stress responses. Cancer Sci. 100:1145–1150. 19486340 10.1111/j.1349-7006.2009.01168.x PMC11159339

[20] Hadaczek P, Siprashvili Z, Markiewski M, Domagala W, Druck T, et al (1998) Absence or reduction of Fhit expression in most clear cell renal carcinomas. Cancer Res. 58:2946–2951. 9679951

[21] Gayrard N, Cacheux V, Iborra F, Mourad G, Argiles A (2008) Cytogenetic studies of 24 renal epithelial tumors with von Hippel-Lindau and fragile histidine triad protein expression correlation. Arch.Pathol.Lab Med. 132:965–973. 18517280 10.5858/2008-132-965-CSORET

[22] Toma MI, Grosser M, Herr A, Aust DE, Meye A, et al (2008) Loss of heterozygosity and copy number abnormality in clear cell renal cell carcinoma discovered by high-density affymetrix 10K single nucleotide polymorphism mapping array. Neoplasia. 10:634–642. 18592004 10.1593/neo.08160 PMC2435001

[23] Moore LE, Jaeger E, Nickerson ML, Brennan P, De Vries S, et al (2012) Genomic copy number alterations in clear cell renal carcinoma: associations with case characteristics and mechanisms of VHL gene inactivation. Oncogenesis. 1:e14. 23552698 10.1038/oncsis.2012.14 PMC3412648

[24] Cohen AJ, Li FP, Berg S, Marchetto DJ, Tsai S, et al (1979) Hereditary renal-cell carcinoma associated with a chromosomal translocation. N.Engl.J.Med. 301:592–595. 470981 10.1056/NEJM197909133011107

[25] Anglard P, Tory K, Brauch H, Weiss GH, Latif F, et al (1991) Molecular analysis of genetic changes in the origin and development of renal cell carcinoma. Cancer Res. 51:1071–1077. 1671754

[26] Strefford JC, Stasevich I, Lane TM, Lu YJ, Oliver T, et al (2005) A combination of molecular cytogenetic analyses reveals complex genetic alterations in conventional renal cell carcinoma. Cancer Genet.Cytogenet. 159:1–9. 15860350 10.1016/j.cancergencyto.2004.09.020

[27] Rein P, Saely CH, Muendlein A, Vonbank A, Drexel H (2010) Serial decline of kidney function as a novel biomarker for the progression of atherothrombotic disease. Atherosclerosis 211:348–352. 20359712 10.1016/j.atherosclerosis.2010.02.031

[28] Liu Y, Shanahan CM (2011) Signalling pathways and vascular calcification. Front Biosci.(Landmark.Ed) 16:1302–1314. 21196233 10.2741/3790

[29] Malliga DE, Wagner D, Fahrleitner-Pammer A (2011) The role of osteoprotegerin (OPG) receptor activator for nuclear factor kappaB ligand (RANKL) in cardiovascular pathology - a review. Wien.Med.Wochenschr. 161:565–570. 21870142 10.1007/s10354-011-0022-7

[30] Ndip A, Wilkinson FL, Jude EB, Boulton AJ, Alexander MY (2014) RANKL-OPG and RAGE modulation in vascular calcification and diabetes: novel targets for therapy. Diabetologia. 10.1007/s00125-014-3348-z 25112376

[31] Wu M, Rementer C, Giachelli CM (2013) Vascular calcification: an update on mechanisms and challenges in treatment. Calcif.Tissue Int. 93:365–373. 23456027 10.1007/s00223-013-9712-z PMC3714357

[32] Firmann M, Mayor V, Vidal PM, Bochud M, Pecoud A, et al (2008) The CoLaus study: a population-based study to investigate the epidemiology and genetic determinants of cardiovascular risk factors and metabolic syndrome. BMC.Cardiovasc.Disord. 8:6. 18366642 10.1186/1471-2261-8-6 PMC2311269

[33] Goek ON, Doring A, Gieger C, Heier M, Koenig W, et al (2012) Serum metabolite concentrations and decreased GFR in the general population. Am.J.Kidney Dis. 60:197–206. 22464876 10.1053/j.ajkd.2012.01.014

[34] Rebholz CM, Astor BC, Grams ME, Halushka MK, Lazo M et al. (2014) Association of plasma levels of soluble receptor for advanced glycation end products and risk of kidney disease: the Atherosclerosis Risk in Communities study. Nephrol.Dial.Transplant. 10.1093/ndt/gfu282 PMC4351358 25147225

[35] Murata K, Baumann NA, Saenger AK, Larson TS, Rule AD, et al (2011) Relative performance of the MDRD and CKD-EPI equations for estimating glomerular filtration rate among patients with varied clinical presentations. Clin.J.Am.Soc.Nephrol. 6:1963–1972. 21737852 10.2215/CJN.02300311 PMC3156428

[36] Drexel H, Amann FW, Beran J, Rentsch K, Candinas R, et al (1994) Plasma triglycerides and three lipoprotein cholesterol fractions are independent predictors of the extent of coronary atherosclerosis. Circulation 90:2230–2235. 7955178 10.1161/01.cir.90.5.2230

[37] Scanlon PJ, Faxon DP, Audet AM, Carabello B, Dehmer GJ, et al (1999) ACC/AHA guidelines for coronary angiography. A report of the American College of Cardiology/American Heart Association Task Force on practice guidelines (Committee on Coronary Angiography). Developed in collaboration with the Society for Cardiac Angiography and Interventions. J.Am.Coll.Cardiol. 33:1756–1824. 10334456 10.1016/s0735-1097(99)00126-6

[38] Alberti KG, Zimmet PZ (1998) Definition, diagnosis and classification of diabetes mellitus and its complications. Part 1: diagnosis and classification of diabetes mellitus provisional report of a WHO consultation. Diabet.Med. 15:539–553. 9686693 10.1002/(SICI)1096-9136(199807)15:7<539::AID-DIA668>3.0.CO;2-S

[39] Saely CH, Koch L, Schmid F, Marte T, Aczel S, et al (2006) Lipoprotein(a), type 2 diabetes and vascular risk in coronary patients. Eur.J.Clin.Invest 36:91–97. 16436090 10.1111/j.1365-2362.2006.01604.x

[40] Rule AD, Larson TS, Bergstralh EJ, Slezak JM, Jacobsen SJ, et al (2004) Using serum creatinine to estimate glomerular filtration rate: accuracy in good health and in chronic kidney disease. Ann.Intern.Med. 141:929–937. 15611490 10.7326/0003-4819-141-12-200412210-00009

[41] Risch L, Saely CH, Neyer U, Hoefle G, Gouya G, et al (2007) Prevalence of decreased glomerular filtration rate in patients seeking non-nephrological medical care—an evaluation using IDMS-traceable creatinine based MDRD as well as Mayo Clinic quadratic equation estimates. Clin.Chim.Acta 378:71–77. 17157286 10.1016/j.cca.2006.10.015

[42] Voight BF, Kang HM, Ding J, Palmer CD, Sidore C, et al (2012) The metabochip, a custom genotyping array for genetic studies of metabolic, cardiovascular, and anthropometric traits. PLoS.Genet. 8:e1002793. 22876189 10.1371/journal.pgen.1002793 PMC3410907

[43] Purcell S, Neale B, Todd-Brown K, Thomas L, Ferreira MA, et al (2007) PLINK: a tool set for whole-genome association and population-based linkage analyses. Am.J.Hum.Genet. 81:559–575. 17701901 10.1086/519795 PMC1950838

